# Potentiality switches and epistemic uncertainty: the Argument from Potential in times of human embryo-like structures

**DOI:** 10.1007/s11019-023-10181-9

**Published:** 2023-10-30

**Authors:** Ana M. Pereira Daoud, Wybo J. Dondorp, Annelien L. Bredenoord, Guido M. W. R. De Wert

**Affiliations:** 1 Department of Health Ethics and Society, Maastricht University, Maastricht, The Netherlands; 2 Department of Medical Humanities, University Medical Center Utrecht, Utrecht, The Netherlands; 3 School for Oncology and Developmental Biology (GROW), Maastricht University, Maastricht, The Netherlands; 4 School for Care and Public Health Research (CAPHRI), Maastricht University, Maastricht, The Netherlands; 5 Socrates chair Ethics of Reproductive Genetics endowed by the Dutch Humanist Association, Amsterdam, The Netherlands; 6 School of Philosophy, Erasmus University Rotterdam, Rotterdam, The Netherlands

**Keywords:** Stem cell-based embryo-like structures, Ethics, The argument from potential, Embryo research, Moral Status

## Abstract

Recent advancements in developmental biology enable the creation of embryo-like structures from human stem cells, which we refer to as human embryo-like structures (hELS). These structures provide promising tools to complement—and perhaps ultimately replace—the use of human embryos in clinical and fundamental research. But what if these hELS—when further improved—also have a claim to moral status? What would that imply for their research use? In this paper, we explore these questions in relation to the traditional answer as to why human embryos should be given greater protection than other (non-)human cells: the so-called Argument from Potential (AfP). According to the AfP, human embryos deserve special moral status because they have the unique potential to develop into persons. While some take the development of hELS to challenge the very foundations of the AfP, the ongoing debate suggests that its dismissal would be premature. Since the AfP is a spectrum of views with different moral implications, it does not need to imply that research with human embryos or hELS that (may) have ‘active’ potential should be completely off-limits. However, the problem with determining active potential in hELS is that this depends on development passing through ‘potentiality switches’ about the precise coordinates of which we are still in the dark. As long as this epistemic uncertainty persists, extending embryo research regulations to research with specific types of hELS would amount to a form of regulative precaution that as such would require further justification.

## Introduction

The culture of both ‘non-integrated’ and ‘integrated’ embryo models from human pluripotent stem cells (hPSCs) marks an important step forward in the refinement of 3D stem cell clusters that recapitulate (aspects of) early embryogenesis in vitro. ‘Non-integrated’ models, such as ‘gastruloids’ (Morris et al. 2020) and ‘axioloids’ (Yamanaka et al. 2023), often lack extraembryonic membranes and are only useful to study segments of early human development. ‘Integrated’ models, such as blastoids (Liu et al. 2021; Yu et al. 2021; Yanagida et al. 2021; Kagawa et al. 2022; Pedroza et al. 2023; De Santis et al. 2023), ‘embryoids’ (Weatherbee et al. 2023), and ‘SEMs’ (Oldak et al. 2023), are more complete and, therefore, presumably (more) capable of resembling the entire conceptus. These clusters can thus differ significantly from each other in terms of cellular origin (i.e., embryonic and/or induced hPSCs), tissue composition (i.e., embryonic and/or extraembryonic membranes), and organizational complexity (e.g., pre- vs. post-implantation stages), and the terms currently in use to describe them are at least as heterogeneous (Rossant and Lam 2021). While terms such as ‘stembryos’ (Veenvliet et al. 2021) and ‘stem cell-based embryo models’ (ISSCR 2021) are becoming more commonly used, we refer to them as human embryo-like structures (hELS) in hopes of more neutrally reflecting their heterogeneity and the degree in which they recapitulate human embryo morphology and functionality.

In addition to increased experimental utility, such as decoupled and bottom-up approaches to human embryology (Posfai et al. 2021), refinement of hELS is driven by the hope it can strike a happy medium between opposite sides of the human embryo research debate: resembling human embryos closely enough to enable important avenues of research while steering sufficiently clear from them to avoid the moral discussions raised by their instrumental research use. Whether this hope is justified, is now prompting moral debate (Denker 2006; Rivron et al. 2018; Sawai et al. 2020; Nicolas et al. 2021; Blasimme and Sugarman 2023). At the crux of this debate is the empirical question of whether current research efforts may lead to improved hELS with a developmental potential akin to ‘natural’ (or: ‘fertilization-based’) human embryos, and the moral question of what that would imply for the acceptability of their use in research. What this would entail in terms of legal implications for researchers working in this field will of course depend on the precise definition of ‘human embryo’ in relevant jurisdictions (Matthews and Morali 2020). Some of these (e.g. Belgium, the Netherlands, and Australia) are actually framed in terms of developmental potential, thus implying that hELS sufficiently similar to natural embryos in this respect are to be regarded as human embryos themselves. This does not follow in countries that still take fertilization as a necessary condition for (clusters of) cells to count as a human embryo (e.g., Spain). Clearly, the adequacy of those definitions is bound to be further challenged as soon as offspring are generated from ELS in other mammalian species.

In this paper, we focus on the moral question and assume a positive answer to the empirical one for the sake of debate. That is, we argue from the particular and—presently—hypothetical scenario in which improved hELS would be able to develop until birth. In the first part of this paper, we revisit the so-called ‘Argument from Potential’ (AfP) in the traditional human embryo research debate. Here, we show that any attempt to justify (more or less restrictive) human embryo protective regulations with reference to the embryo’s intrinsic moral status must require an appeal to the AfP, i.e., the argument that early human embryos deserve (some degree of) protection because they have the potential to develop into mature human beings. Moreover, we show that the AfP can best be understood in terms of a spectrum along which different versions are possible, each with different implications for the degree of protection it can confer. In the second part of this paper, we synthesize the findings of the foregoing sections to illuminate difficulties with applying (versions of) the AfP that have so far received little scholarly attention. We first argue that, while the validity of the AfP has been criticized in light of the very developments leading up to the creation of hELS, these critiques remain inconclusive: it is possible that the AfP can be maintained not only with regard to human embryo research but also with regard to research with hELS. Next, we show that maintaining the AfP will nonetheless still require further characterization. At present, it is unclear how the argument should apply because, contrary to human embryos, it is much more challenging to identify whatever “switches” could be responsible for the inception of active potential in hELS.

### The AfP in the traditional human embryo research debate

With human embryos becoming available for research purposes due to developments in in vitro fertilization (IVF) and related biotechnologies at the end of the 20th century, came the question of whether and, if so, under what conditions, such research would be ethically and legally acceptable. This question stemmed from the widely shared intuition that human embryos were somehow morally special when compared to other (non-)human cells. Not because their scarcity made them valuable research material that should be used prudently, but because many viewed them as entities “toward which moral agents have, or can have, moral obligations” (Warren 1997, 3): entities with (intrinsic) *moral status*, the research use of which, if acceptable, would require due diligence.

### Accounting for the special moral status of the embryo

What could give human embryos a moral status that would restrain their instrumental use in scientific research? The fact that they are living entities would certainly not suffice to make this claim, as it would include far too much. A better candidate would be sentience: it is because sentient animals have needs and interests—including an interest in not experiencing pain or discomfort—that their instrumental research use is limited to important research aims only (proportionality), specifically those that cannot be achieved through morally less sensitive means (subsidiarity) (LaFollette 2011). What would sentience entail for human embryo research? The wish to steer clear from conducting research at developmental stages where human embryos might feel pain has certainly played a role in the Warnock (Warnock 1984) Committee’s recommendations leading to the influential 14-day rule, which prohibits the research use of human embryos beyond fourteen days post-fertilization and has since been adopted internationally (Hyun et al. 2016; Pera 2017). However, it also seems clear that, at least as far as sentience is concerned, the 14-day rule is overcautious. As it presupposes a degree of brain development that can only be acquired at fetal stages (Lowery et al. 2007; Bellieni 2019; Derbyshire and Bockmann 2020), it seems fair to conclude that there is no reason to constrain human embryo research on this basis. Moreover, if sentience were all that counts and it does not stand in the way of using animals (or animal embryos) in research, it is unclear why its emergence would stand in the way of the similar use of human embryos.

Of course, it is precisely the fact that human embryos are human that many intuitively regard as making a moral difference. Could their humanity be a convincing ground for according them a (special) moral status that animals or embryos of other species lack? Most people would indeed agree that human beings have a higher moral status than other sentient non-human animals. This is reflected in the fact that, whereas sentient non-human animals might justifiably be used as research material under procedural and material conditions, similar use of human beings would be morally unacceptable and legally forbidden in all countries with human rights based research legislation. They may of course participate as research *subjects* based on their informed consent in studies approved by ethics review committees, but that is precisely what marks the difference with using them as research *material*. Why is it that human beings are thought to have this higher (or full) moral status that (as famously phrased by Kant (Kant 1998) forbids their use as ‘mere means’? In order to avoid charges of circularity and speciesism, we must have a reason beyond the mere fact that they are human.

It is here that the concept of personhood comes into view. Typical human beings are not just sentient animals; they are also *persons*. On John Locke’s classical definition, a person is “a thinking intelligent being, that has reason and reflection, and can consider itself as itself, the same thinking thing, in different times and places” (Locke 1997, 302). Explaining the moral relevance of the concept, Taylor succinctly states, “a person is a being who can be addressed and who can reply” (Taylor 1958, 97). That is to say, a person deserves to be respected as a moral subject, whereas sentient non-persons can only enter the moral community as moral objects. Characteristics widely associated with personhood can be listed as including “autonomy, rationality, self-awareness, linguistic competence, sociability, the capacity for intentional action, and moral agency” (DeGrazia 2005, 6). However, as the concept defies precise definition, it remains a matter of controversy which and how many of the relevant characteristics a being should have in order to qualify as a person. For our purposes, we do not need to go into those debates. It suffices to note that as human embryos have none of these characteristics, they are certainly not persons in the Lockean sense.^1^ The claim that they might nonetheless deserve (some degree of) protection for their own sake (i.e., intrinsic moral status) would thus still require a further argument, for which the AfP is a plausible candidate (Stone 1987).

### Full and limited versions of the AfP

**Fig. 1:**
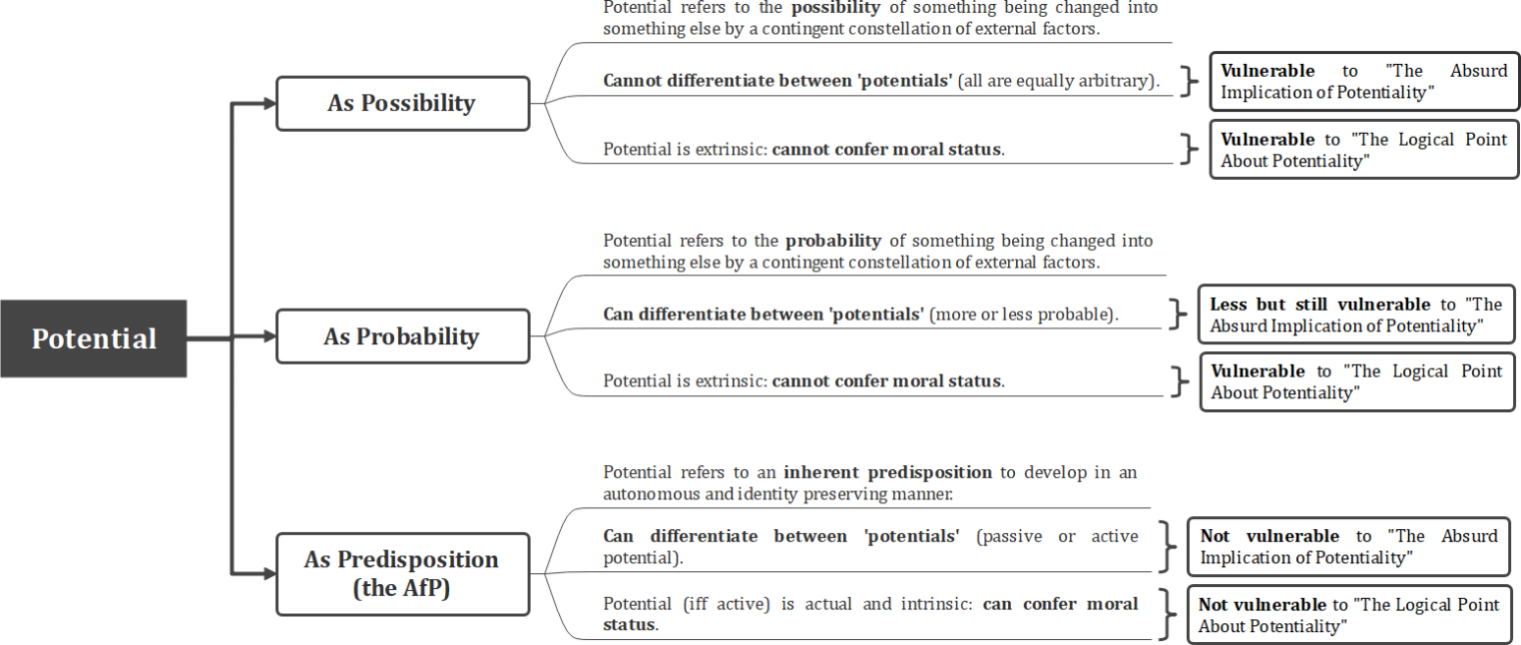
Chart of possible differences in AfP positions with regard to the moral bearing and onset of active potential

The intuitive appeal of the AfP lies in how it links the present embryo and later paradigmatic person or, in other words, in how it accounts for both the *continuity* and *discontinuity* between what the embryo currently is and what it has the potential to become in the future. Nonetheless, advocates of the argument can and do differ with regard to which of these aspects they emphasize, and that can in turn lead to different views about the moral status of human embryos and the acceptability of their use in research (Fig. 1). Those who emphasize the continuity aspect, generally take the AfP to entail that human embryos have the same (‘full’) moral status as human persons, which would imply that they should not be used as mere research material. An example of this stance can be found in the dissenting position to the Warnock Report by three of its Committee members, according to whom

“… the embryo has a special status because of its development to a stage at which everyone would accord it the status of a human person. It is in our view wrong to create something with the potential for becoming a human person and then deliberately to destroy it”. (Warnock 1984, 90)

Another example can be found in the work of Reichlin, who argues even more explicitly, “human embryos must be treated as persons, since personhood is their destiny” (Reichlin 1997, 7). We will refer to these interpretations of the argument as the ‘Full Moral Status Versions of the AfP’ or ‘Full AfP’, for short.

By contrast, those who emphasize the discontinuity aspect, regard the fact that human embryos are only *potential* persons to mean that any moral status derived from this potential must be limited as compared to that of *actual* persons. This is often spelled out in ‘gradualistic’ terms, capturing the widely held intuition that the claim to consideration that human embryos (and human fetuses) have is initially weak, but that its strength increases with later stages of development (Poplawski and Gillett 1991; Kant 1998; Heinemann and Honnefelder 2002; Álvarez-Diaz 2007). Since this view is compatible with allowing human embryo research under conditions of proportionality and subsidiarity until later developmental stages (Heinemann and Honnefelder 2002; Pugh 2014), it can typically be found in policy documents accompanying more or less liberal embryo research regulations. The Explanatory Memorandum to the Dutch Embryos Act, for example, states that “what makes an embryo worthy of protection (…) is its potential to grow into a human being” (Tweede Kamer der Staten-Generaal 2001, 49), but it does not suggest that this protection should go beyond setting certain limits that still allow important research to proceed. As explained in an earlier report from the Dutch Health Council, the human embryo’s moral “value is on the one hand determined by its potential to grow into an individual (…), but on the other hand by the fact that this development has only just begun”, thus making it conceivable that “other values and interests outweigh [its] worth” (Gezondheidsraad 1986, 74–75). We will refer to these interpretations of the argument as the ‘Limited Moral Status Versions of the AfP’, or ‘Limited AfP’, for short.

### Possibility, probability, and (active) potential

The AfP has also met with criticism. As conveyed by what Feinberg coined “the logical point about potentiality” (Feinberg 1992, 51), nothing follows for the embryo’s actual moral status from the fact that it might change into a future being whose moral status will then be uncontested. Moreover, as Harris (Harris 1985) and others (Singer and Dawson 1988; Warnock 1987) have argued, the argument would require us to protect much more than only human embryos:

“To say that a fertilised egg is potentially a human being is just to say that if certain things happen to it (like implantation), and certain other things do not happen (like spontaneous abortion), it will eventually become a human being. But the same is also true of the unfertilised egg and the sperm.” (Harris 1985, 11).

The subsequent and often reiterated ‘absurd’ implication that human gametes should then also be afforded moral status is meant to bring home that the AfP is better abandoned. These authors converge in that they interpret the concept of potential as the mere possibility of something being changed into something else by a contingent constellation of external factors (or, ‘arbitrary things happening to it’). If that is what the AfP is taken to refer to, then it is indeed difficult to see how it could retain any moral bearing.

Other authors have argued that the embryo’s potential should be understood in terms of probability, rather than mere possibility (Noonan 1970; Engelhardt 1986). Would this interpretation save the AfP from the foregoing criticisms? Perhaps it could take the brunt of the ‘absurd implication’. If greater probability of maturing into a human being were to mean greater moral status, then it would be possible to draw moral distinctions based on the entity’s (i) developmental stage (early vs. late), (ii) circumstantial environment (in vivo vs. in vitro), (iii) creation purposes (research vs. reproductive), and indeed (iv) organizational level (organisms vs. reproductive cells). However, it would still run up against Feinberg’s logical point—i.e., the fallacy of deducing actual moral status from what the embryo is only in potential (Feinberg 1992).

**Fig. 2:**
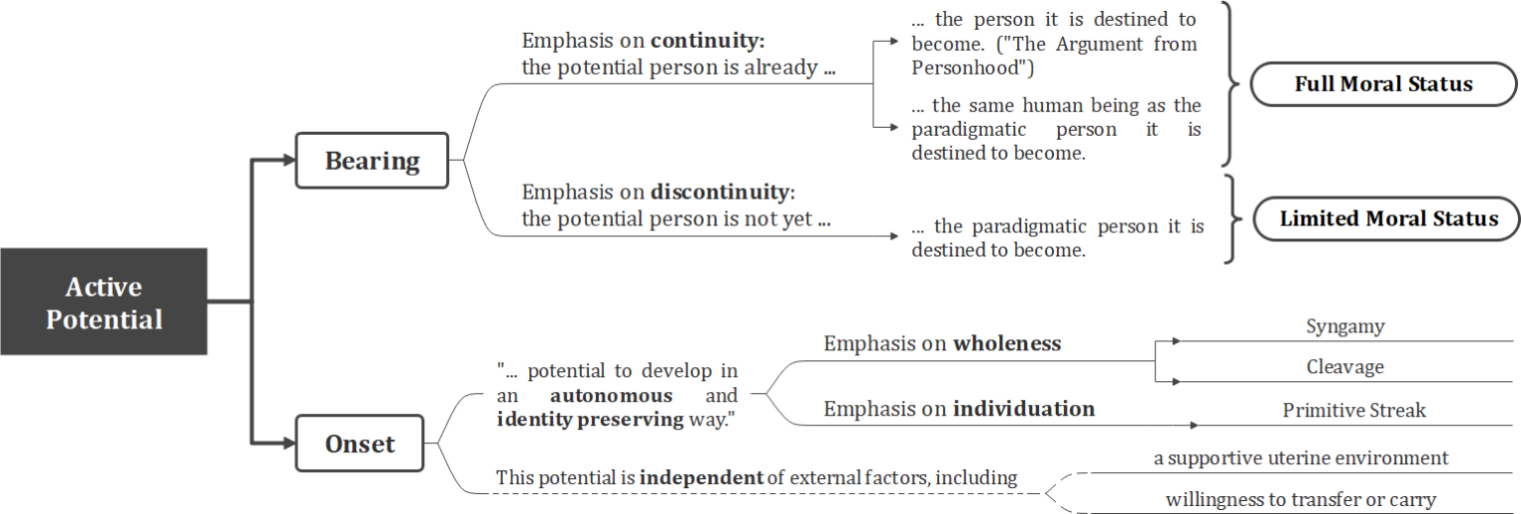
Chart of how potential could be interpreted differently and what these differences would imply for attributing moral status on that basis

Advocates of the AfP, however, remain unconvinced by these criticisms, which they counter argue to be straw man fallacies (Stone 1987). Contrary to what critics purport, the argument is not about personhood being an empirically possible (or probable) outcome, but rather about it being the outcome towards which the embryo is intrinsically predisposed (DeGrazia 2012) (Fig. 2). As stated by Reichlin, the idea is that

“… the embryo’s development does not depend on external causes, rather on an inherent teleology that only demands certain environmental factors to be displayed: the embryo has itself the potential for full personhood, and does not receive it from outside.” (Reichlin 1997, 7).

In the literature, this contrast in ‘types of potential’ is commonly explained in terms of Aristotle’s distinction between *passive* and *active* potential (Aristotle 1998), or Buckle’s *potential to produce* and *potential to become* (Buckle 1990). Whereas the former notion is used to refer to the possibility of an entity changing into something else by virtue of external causes, the latter is used to denote the *autonomous* and *identity preserving* development that can establish a relationship between what the entity currently is and what it is intrinsically destined to become. On this understanding of the AfP, there is no logical gap between potential and actual moral status because the potential to become persons must already be an actual characteristic of the developing entity. As remarked by DeGrazia (DeGrazia 2012), this makes it a more cogent argument than its critics tend to suggest. Moreover, since gametes are separate organismal unities, they cannot be identical with the person(s) that arise from them. This means that the relation between gametes and future persons can only be one of producing, rather than becoming, and that the charge that the AfP would have the ‘absurd implication’ of also applying to the unfertilized egg and sperm therefore fails.

### Different views on the moral bearing of active potential

Both Full and Limited versions of the argument allow for different ways in which they explain the moral bearing of active potential, depending (again) on whether they emphasize the continuity or the discontinuity in the relationship it establishes (Fig. 1).

Full versions can come in one of two variants. According to the version of the Full AfP that might just as well be referred to as the ‘Argument from Personhood’, the embryo is already the person it is destined to become in a more full-fledged sense. On this view, the later development of Lockean properties (such as self-consciousness, rationality, and moral agency) confirms, rather than establishes, the personhood that characterizes human beings at all stages of development (Lee 2004). An alternative version of the Full AfP avoids equating personhood with species membership and departs from the notion that the embryo is the same human being as the paradigmatic person it is naturally destined to become. According to this view, human embryos have a strong interest in realizing their active potential, and that interest grounds a right to care and protection that is of equal strength to that of full-fledged persons (Stone 1987). As observed by Steinbock (Steinbock 2011), this reasoning is implicit in Marquis’ ‘Future-Like-Ours Argument’ (Marquis 1989) because only beings destined to become persons could have valuable futures like ours.

Limited versions of the AfP also build on the notion of active potential as an actual and morally relevant characteristic of the developing entity, but without affording them full moral status on that basis (Heinemann and Honnefelder 2002). One way of explaining this lower moral status has been suggested by DeGrazia. Given that, prior to the emergence of sentience, there can be no psychological unity that would link fetuses (or embryos) with the paradigmatic persons they will become at future stages of their lives, whatever interest they presently have in realizing their potential can only be weak (DeGrazia 2012). While DeGrazia does not commit himself to the view that pre-sentient fetuses (or embryos) could have such an interest, the fact that he nevertheless presents it as a defensible position connects with his view that we are essentially animal organisms, rather than (embodied) minds. Clearly, this is also the position of those adhering to (Full or Limited versions of) the AfP in the human embryo research debate (Alvarez Manninen 2007). On the opposite metaphysical view, as defended by McMahan (McMahan 2002), the earliest possible stage at which a human being with active potential for paradigmatic personhood can be present is from 20 weeks of gestation onwards. Given that the fetus’s time-relative interest in realizing this potential can only be weak (a point on which DeGrazia (DeGrazia 2012) and McMahan (McMahan 2002) converge), what follows is a version of the Limited AfP whose relevance is restricted to debates about the moral status of developed fetuses (and subsequent implications for late abortion and fetal interventions), not human embryos.

### Different views on the onset of active potential

While advocates of the AfP thus agree that there can be no active potential prior to fertilization, there are different views as to the point from whence it can be acquired (Fig. 1). Some see completion of fertilization as the obvious onset for active potential, as from syngamy onwards there is a living organism that is internally programmed “to develop in the species-specific way” (Jochemsen et al. 2004, 88). The emphasis here is on autonomous development and organismic wholeness. Others doubt whether that can be enough to identify the pre-implantation embryo with the individual person(s) it may grow into. A first reason for this is that most cells of the pre-implantation embryo will contribute to the formation of extraembryonic tissues, rather than to the formation of the ‘embryo proper’, which only emerges with the process of gastrulation at around two weeks of development. A second and more important reason is the possibility for fusion or splitting occurring, which many take as evidence that embryonic development cannot be identity preserving prior to these stages (Curran 1979; Buckle 1990; Persson 2003). The emphasis here is on individuation as a further condition for the onset of active potential.

For authors that maintain that active potential can only be ascribed to (post-)gastrulation embryos, the Full AfP could serve as an argument for underscoring the 14-day limit (and the development of the primitive streak, specifically) as a cut-off point for research, whereas the Limited AfP would only require imposing certain conditions on research beyond that stage. However, neither would provide convincing grounds for forbidding or regulating research with pre-gastrulation embryos. By contrast, authors who do not share this view on identity preservation could maintain that active potential (and the ensuing implications, which would again depend on whether they defend Full or Limited versions of the AfP) also applies to pre-gastrulating embryos. Reichlin, for example, maintains that, “the attainment of indivisibility (…) perfects the individuality of the embryo, but (…) does not show by itself that a new individual is present only at this stage” (Reichlin 1997, 21).

### The AfP in the current human embryo(-like) research debate

In this paper, we assume that the ability to model embryonic morphology and functionality in clusters of (induced) hPSCs raises the prospect of further refinements resulting in hELS with a potential to develop into human beings. What would this scenario entail for their moral status and for how we should treat them in research? In terms of the foregoing discussion, it would seem that the crucial question is whether this potential should be qualified as passive or active. If passive, then it would be a matter of mere possibility and therefore not establish moral status. If active, then we would be dealing with cells that are at least morally equivalent to human embryos. For several authors, however, the developments prompting this very question only come to show that there can be no such thing as active potentiality in developmental biology, and that the cellular convertibility increasingly demonstrated by experiments in the field should be seen as the finishing blow to the AfP.

### Ongoing debate about the validity of the AfP

Before hELS could be reported, Stier and Schoene-Seifert had already argued that the AfP could no longer be sustained in light of contemporary advancements in developmental biology (Stier and Schoene-Seifert 2013). Their argument referred specifically to experiments with tetraploid complementation in mice, which presumably showed that a tetraploid environment could trigger induced PSCs (iPSCs) injected into aggregate embryos to convert back into a state of totipotency, ultimately generating offspring. Assuming the same would apply in humans, Stier and Schoene-Seifert argued that these insights represented a fatal variant of the absurd implication. After all, if adult cells can be induced back into a state of totipotency and ultimately develop into mature human beings, then this ability must be innate to all human cells and require only that the right environmental triggers are ‘switched on’. Piotrowska has presented similar arguments (Piotrowska 2020, 2021). In her view, the belief that human embryos develop autonomously into mature human beings is “an artifact of our pre-biotechnological past” (Piotrowska 2020, 175) that needs to be set aside. Keeping it would ignore the fundamental influence of an instructive (uterine) environment in the realization of the human embryo’s potential to develop into a human being, rather than, for example, into a tumor.

The view that current science has no place for the notion of active potential has itself invited criticism, however. In his commentary to Stier and Schoene-Seifert, Hyun (Hyun 2013) argued against stretching the implications of tetraploid complementation experiments in mice. Apart from the fact that these findings might not translate to human embryos, they fail to provide evidence for the particular point Stier and Schoene-Seifert try to make. Rather than showing that developmental potential could be triggered in individual iPSCs and that it therefore only needs to be released by appropriate external switches, the experiments seem to show that it can only emerge when clusters of cells interact as a unitary whole, which is not at odds with the notion of autonomous development. Although Hyun is explicitly not seeking to defend the AfP, which he interprets as necessarily and problematically implying that early human embryos would have full moral status on that basis (i.e., the Full AfP), his observations can be taken to support the AfP’s central distinction between active and passive potential. A more categorical defense of this distinction in the current debate can be found in the work of Denker (Denker 2015, 2021). Against the view that the signals for ‘symmetry breaking’—which are crucial for germ layer development and body plan formation in mammalian embryonic development—come from the uterine environment, he argues that there is increasing evidence that these signals in fact emanate from the embryo itself (Denker 2021). In other words, symmetry breaking is what the embryo autonomously does, rather than what happens to it. For Denker, these insights add credence to the view that “mammalian embryos are (…) complete developmental systems, possessing active (not just passive) developmental potential” (Denker 2021, 9). Moreover, he argues, the fact that this self-organizing capacity has also been reported in hELS suggests that these structures may also acquire the autonomous (or active) potential relevant for moral status. Which, ethically, he further suggests should be considered a “quantum leap with regard to the dignity to be ascribed to a colony of stem cells, moving it into the same ethical category as an embryo of that stage” (Denker 2021, 9).

While the AfP remains empirically and analytically contested, this ongoing debate suggests that claiming its obsolescence in light of current insights in developmental biology might be premature. As it stands, the AfP may continue to play a role not only where the ethics and regulation of traditional human embryo research are concerned, but now also in that of research involving three-dimensional clusters of human stem cells (i.e., hELS). Clearly, active potential cannot be ascribed to hELS that model exclusively extraembryonic tissues (which should instead be referred to as extraembryonic *organoids*), but the argument might hold for structures that (also) model embryonic lineages.

### Potentiality switches

If we suppose that certain hELS have active potential, this must mean that it is possible to control its emergence in a petri dish; i.e., that active potential can be ‘switched on’ by altering the composition of a cluster of cells that previously only had passive potential. This is not entirely new, as for those holding the view that the active potential of natural embryos starts at conception, the process of fertilization can also be seen as a controllable potentiality switch. For those who consider gastrulation to be (necessary for) the onset of active potential, allowing the embryo to undergo that process might also be considered such a switch.

In human embryos, it is clear where those switches are and what they imply for moral status (depending of course on whether one holds Full or Limited versions of the AfP). In hELS, however, it is far from clear how these switches can be identified: at what point in their development can hELS acquire active potential? This question is important because, as long as we are in the dark about the precise coordinates of the relevant switches, we cannot tell whether the material that researchers are working with may or may not be regarded as entities with (some degree of) moral status. In this connection, Denker sees an urgent need to elucidate under what

“… conditions a group of stem cells may start the way to autonomy in the sense of gaining independence of pattern formation from outside signals, how this specific state of developmental autonomy can be detected, and how the process can be controlled” (Denker 2021, 9).

Here again, for active potential to be present, development must be both autonomous and identity preserving. Denker seems to regard this as one and the same potentiality switch that may change the moral status of hELS already at pre-gastrulation stages. As an advocate of the Full AfP, he cautions therefore that whatever the benefits for (clinical) science, experiments “leading to the formation of blastocyst-like or gastruloid constructs, should (…) for ethical reasons” (Denker 2021, 9) not be done with human (but rather non-human primate) stem cells. However, for those holding that an identity preserving development cannot begin at pre-gastrulation stages, the emergence of autonomy at those stages is at most a precondition for any later passive-to-active potentiality switch. Blastoids (like blastocysts) are not potential persons in their view.

An interesting question that illustrates the difficulties of applying the AfP in the current debate is whether hELS that contain the cells typically found in the embryo proper but that are nonetheless incomplete in the sense of lacking (those that will form) extraembryonic ones, should therefore be regarded as lacking active potential. Gastruloids, for example, may be considered similar to the embryo proper in terms of how they form the derivatives of the three germ layers and undergo axis-formation in vitro (Moris et al. 2020) despite lacking extraembryonic tissues. Sawai et al. (Sawai et al. 2020), who raise this question, qualify these structures as having only passive potential because of how they would require hypothetical scaffolding and culturing technologies in order to develop further. These authors seem to conceive the use of these additional technologies as ‘transformative’ (i.e., in the sense of representing a switch from passive to active potential) and, based on what is currently known about the importance of (timely) cellular interactions for faithful morphology and functionality, there seems to be good enough reasons to think this is the case.

This does not take away that, if we suppose that it could become possible to also substitute the signaling provided by those interactions with technological interventions, those interactions too could be conceived as merely supportive. Perhaps these (future) technologies would then not represent the change, but the ‘help’ that those structures need in order to develop into mature human beings. How would this help be qualitatively different from the help in vitro embryos need (e.g., transfer to an appropriate environment) in order to realize their active potential? As the active potential of in vitro embryos does not depend on whether they are transferred to a womb, there might be a case for reasoning that a lack of extraembryonic membranes would similarly not prevent gastruloids from having active potential. Without aiming to either refute or defend this reasoning, it is worth pointing out that it could lead to noticeable differences in how to interpret its moral implications. Since not all AfP advocates would consider identity preserving potential to be present at stages where fission or fusion may still occur, this could, for example, be taken to imply that gastruloids have a more secure claim to moral status than more complete hELS (e.g., human blastoids) and, indeed, human embryos (e.g., early human blastocysts), at pre-gastrulation stages.

### Dealing with uncertainty

Talk of ‘potentiality switches’ remains of course hypothetical as long as we do not know whether (improved) hELS would actually be able to develop into a mature human being. Noting that ethical concerns (including the ban on reproductive cloning) prevent us from doing the relevant experiments and that no offspring have so far been born from mammalian ELS^2^, the question of how we should deal with this epistemic uncertainty remains in the meantime. At present, there seems to be a growing tendency to argue for precaution. Sawai and colleagues, for example, propose a pragmatic consistency approach in which research with hELS that have every component of natural human embryos (including extraembryonic tissues) should be regulated in the same way as research with natural human embryos at similar stages (Sawai et al. 2020). This could practically imply restricting research with human blastoids as we would with human blastocysts (as currently done in Australia (Australian Government 2021)), even though it remains scientifically disputed whether these models in fact resemble human blastocysts in every respect (Posfai et al. 2021). The scholarly consensus reflected in the Updated Guidelines of the International Society for Stem Cell Research (ISSCR 2021) is another example of similarly precautious conclusions. While presented as pragmatic, precautionary approaches such as these are arguably better defended on the basis that human embryos or equivalent entities deserve (some level of) protection for their own sake than on the view that they do not. Without a claim to moral status, there would be little to outweigh the price of refraining from important avenues of hELS research or for imposing regulative burdens on researchers other than a wish to avoid public concerns or sensitivities. Of course, the case for such regulative precaution would even then require further justification, precisely in view of its proportionality (Steel 2014). While entering into this debate would be beyond the scope of our paper, it is important to note that different versions of the AfP come with different understandings of the moral price of failing to protect hELS that may well be embryos and, therefore, also of the proportionality of treating them as embryos in the light of epistemic uncertainty.

As a specific precautionary measure, it has been suggested that one might think of genetically modifying hELS so as to suppress development beyond a certain point and to ensure that the entity could not possibly grow into a mature human being (Rivron et al. 2018). This is sometimes referred to as building in ‘suicide genes’. Of course, if the modification is built in at stages where active potential may already exist, this should be regarded as an active-to-passive potentiality switch, which as such would not serve to lessen AfP-based concerns. Building in such a switch would clearly be unacceptable on Full versions of the AfP. On Limited versions, it could be argued that meeting the subsidiarity requirement would be difficult, given that those genes could also have been built-in at whatever stages clearly precede the possible emergence of active potential. But then indeed: what about a pre-emptive modification at those earlier stages?

One might think of doing this modification in the separate hPSCs used to create hELS, thereby ensuring that the resulting clusters of cells do not develop beyond a certain point or that they fail to reach essential milestones. Whether this strategy avoids raising ethical concerns based on the AfP depends on whether it would prevent the emergence of active potential and ensuing moral status, however, and not all advocates of the Full AfP can be expected to agree that it would. Those adhering to the version of the AfP that we have earlier referred to as “the Argument from Personhood” might follow the reasoning of Doerflinger (The President’s Council on Bioethics 2004), the representative of the U.S. Catholic Bishops’ Conference, in the earlier ‘altered nuclear transfer’ debate. This debate, which can be seen as a precedent to our discussion, centered on the idea of combining cloning and genetic modification in order to create non-viable human embryos as a supposedly morally neutral source of human embryonic stem cells for research and therapeutic purposes (Hurlbut 2005). Doerflinger did not buy into this. According to him, if those non-viable human embryos would only differ from normal human embryos in terms of not being able to develop beyond a certain point, then the proposal would simply amount to creating human persons with a deliberately shortened life span (The President’s Council on Bioethics 2004). Piotrowska refers to this precedent as a further illustration of the problems she sees with the AfP: apparently, its advocates would even have us protect entities without any potential to grow into a mature human being (Piotrowska 2021). However, that seems too strong of a conclusion, as it ignores the specific reasoning underlying this version of the AfP and how it differs from all other AfP versions, namely that it sees active potential as a confirmation of personhood, rather than as a condition for attaining it. On all other accounts of the AfP, pre-emptively building in developmental roadblocks may well entail that there is no potential for personhood from the start and, therefore, no moral status. For this to hold true, however, it is essential that these roadblocks obstruct the potential for further development ‘from within’, rather than merely frustrating it ‘from without’. Cutting short essential developmental pathways in (the precursor cells of) the embryo proper would seem to fulfill this requirement, but it is less clear whether the same would apply to cutting-off genes that only affect the capacity to implant. In light of the foregoing discussion on whether lack of extraembryonic tissues would stand in the way of ascribing active potential to gastruloids, one might similarly argue that blastoids in which the genes for implantation have been suppressed would ultimately be comparable to in vitro embryos to which transfer to a uterus has been denied. If the latter does not stand in the way of having active potential, then the same could perhaps also be said with regard to the former. Building-in suppressive genes that would instead affect the development of the embryo proper, might be regarded as a meaningful precautionary approach by most advocates of the AfP. Obviously, the need for such measures will be more strongly felt by those adhering to (the remaining version of) the Full AfP, rather than to Limited versions. For the former, the case for building-in suppressing genes is a matter of allowing hELS (and human embryo) research in the first place. For the latter, it may still be important as a matter of avoiding the restrictions and regulative burdens of their use in research.

### Concluding remarks

Where human embryo research is allowed, it is often only permitted under conditions of proportionality and subsidiarity, and only up to 14 days of development post-fertilization (or the development of the primitive streak if that comes first). We suggest that if these regulations are meant to reflect the view that human embryos have intrinsic moral status and, therefore, deserve (some degree of) protection, they can be accounted for in terms of the AfP. This can clearly not be versions of the AfP that ascribe full moral status to human embryos from conception, as that would require forbidding destructive human embryo research altogether. The 14-day rule could be accounted for in terms of the Full AfP in its ‘from gastrulation’ version, but this would undermine the case for an AfP-based justification of the regulatory conditions imposed on research with pre-gastrulation human embryos. This tension would not arise on the widely held interpretation of the 14-day rule as a pragmatic rather than principled line. Shifting that line to, for example, 28 days, or making it flexible, as recently proposed by the ISSCR (ISSCR 2021), would be compatible with both versions of the Limited AfP.

While the AfP can thus be seen as underlying current embryo research regulations, it has always been contested. This criticism has gained further strength with developments in hELS-research, purportedly showing that there is no such thing as ‘active potential’. If true, this would not only undermine the case for building regulations around the notion that hELS with significant developmental potential should be treated differently from those without, but also put current regulations of traditional embryo research on a much weaker footing than if the AfP would apply. This matters because limiting important research in these fields is less easily justified if it cannot be done in terms of respecting the intrinsic moral status of human embryos and functionally equivalent hELS.

As this debate is ongoing, it seems premature to relegate ‘active potential’ to the dung heap of the history of philosophy. But if, as we have suggested for the sake of argument, the AfP can be maintained, it is not immediately obvious how it should be applied to the new field of hELS research. As we have suggested, the problem with determining active potential is that this depends on development passing through—what we have referred to as—‘potentiality switches’ about the precise coordinates of which we are still in the dark. Whether this epistemic uncertainty should be seen as calling for regulative precaution, is a matter for further debate. A specifically interesting measure is the proposal to suppress developmental potential pre-emptively beyond a certain point. While this would not be acceptable according to what we have called the ‘from Personhood’ reading of the Full AfP, it would seem to be an acceptable strategy in principle on other versions of the AfP, notably when it involves obstructing developmental pathways that would affect the development of a functionally organized embryo proper. In principle, given that the proportionality of such measures (also in terms of how they might limit important research avenues) should be part of a comprehensive ethical evaluation.

Interestingly, commentators who have argued that the AfP can no longer be maintained in times of hELS, do not tend to conclude that it would thus also be impossible to ascribe moral status to human embryos or functionally equivalent hELS. For instance, Piotrowska (Piotrowska 2020, 2021) and others (Aach et al. 2017) have proposed regulators do away with the whole idea that developmental potential might matter morally and, instead, simply consider which morally relevant features may arise in hELS. However, short of the actual capacity to feel pain (which is not realistic at embryonic stages), it is unclear what other features we could think of (e.g.: primitive streak, heartbeat, neurological substrates, etcetera) whose moral significance would not tacitly depend on the AfP.

## Footnotes

1 The adjective is meant to acknowledge the different account of personhood as upheld by religious authors from traditions with Aristotelian roots, including Roman Catholic moral theologians and writers with a Jewish or Islamic background. They argue that personhood is given, not with any specific characteristics, but with the unity of a human body and a rational human soul **(**Boyle 1991**)**. In this view, human embryos are persons from the moment of their ‘ensoulment’. As Jewish and Islamic authors (and indeed many Catholic theologians) still do, the pre-nineteenth century Church upheld a theory of ‘delayed ensoulment’ (McComick 1991). Here, it followed Aquinas’ (and Aristotle’s) view that as the rational soul must be “the act of an organic body”, its infusion can only take place after the initial development of the organs necessary for rational action, leading to a belief in ensoulment at 40 days of development, or even later. By contrast, the present Roman-Catholic position is that it cannot be excluded that ensoulment takes place at conception, and that the zygote should therefore already be treated as a person **(**Pope John Paul II 1987**)**. Clearly, only this ‘immediate ensoulment’ variant of the religious account of personhood stands in the way of conditionally allowing research using human embryos at early stages of their development. As stated by John-Paul II, “the mere probability that a human person is involved would suffice to justify an absolutely clear prohibition of any intervention aimed at killing a human embryo” **(**Pope John Paul II 1995, **#60)**. Later in this paper, we suggest that the view that the embryo is already a person prior to manifesting any of the characteristics of personhood can be seen as a radical instance of (the Full version of) the AfP.

2 This research is ongoing, with recent studies having reported the successful implantation of mouse **(**Tarazi et al. 2022) and macaque **(**Li et al. 2023) embryo models. At the time of writing, however, none has been able to generate offspring.
